# Detection of copy number variation from array intensity and sequencing read depth using a stepwise Bayesian model

**DOI:** 10.1186/1471-2105-11-539

**Published:** 2010-10-31

**Authors:** Zhengdong D Zhang, Mark B Gerstein

**Affiliations:** 1 Department of Genetics, Albert Einstein College of Medicine, Bronx, NY 10461, USA; 2 Department of Molecular Biophysics and Biochemistry, Yale University, New Haven, CT 06520, USA; 3 Interdepartmental Program in Computational Biology and Bioinformatics, Yale University, New Haven, CT 06520, USA; 4 Department of Computer Science, Yale University, New Haven, CT 06520, USA

## Abstract

**Background:**

Copy number variants (CNVs) have been demonstrated to occur at a high frequency and are now widely believed to make a significant contribution to the phenotypic variation in human populations. Array-based comparative genomic hybridization (array-CGH) and newly developed read-depth approach through ultrahigh throughput genomic sequencing both provide rapid, robust, and comprehensive methods to identify CNVs on a whole-genome scale.

**Results:**

We developed a Bayesian statistical analysis algorithm for the detection of CNVs from both types of genomic data. The algorithm can analyze such data obtained from PCR-based bacterial artificial chromosome arrays, high-density oligonucleotide arrays, and more recently developed high-throughput DNA sequencing. Treating parameters--e.g., the number of CNVs, the position of each CNV, and the data noise level--that define the underlying data generating process as random variables, our approach derives the posterior distribution of the genomic CNV structure given the observed data. Sampling from the posterior distribution using a Markov chain Monte Carlo method, we get not only best estimates for these unknown parameters but also Bayesian credible intervals for the estimates. We illustrate the characteristics of our algorithm by applying it to both synthetic and experimental data sets in comparison to other segmentation algorithms.

**Conclusions:**

In particular, the synthetic data comparison shows that our method is more sensitive than other approaches at low false positive rates. Furthermore, given its Bayesian origin, our method can also be seen as a technique to refine CNVs identified by fast point-estimate methods and also as a framework to integrate array-CGH and sequencing data with other CNV-related biological knowledge, all through informative priors.

## Background

Stable but not static, the DNA of human genome is subject to a variety of heritable changes of different types, which significantly contribute to the phenotypic differences of individuals in human populations. In addition to the single nucleotide polymorphisms (SNPs), these genetic changes also include the chromosomal structural variations, such as insertions, deletions, duplications, inversions, and translocations, on various genomic scales. Recent studies showed that insertions, deletions, and duplications of DNA segments of 1 kb or longer in the genome-- collectively referred to as the copy number variants (CNVs)--occur at a much higher frequency than previously expected [1-4]. A recent global study of CNVs in the human genome showed that the regions of CNVs covered more nucleotide content per genome than SNPs [1]. It is now widely believed that CNVs are as important as SNPs and other small genomic changes in their contribution to the phenotypic variation in human populations.

Currently, unbalanced structural variants can be experimentally identified by methods based on microarray technology, polymerase chain reaction, or DNA sequence comparison. Array-based method is a natural, high-throughput extension of the comparative genomic hybridization (CGH) analysis, which was originally developed as a method to reveal any regions of allele loss or aneuploidy by fluorescence microscopy [5]. High-density oligonucleotide microarrays, which offer high genomic resolution, have been used in several recent array-CGH studies [4,6,7]. The last several years have seen rapid advancement in the field of sequencing technology. Novel methods [8-10] are being developed to reduce the cost and increase the throughput by generating massive amounts of sequence that can be aligned to the genomic reference. This development has made it possible to resequence whole genomes from multiple individuals.

Indeed, a major sequencing project, the 1000 Genomes Project, has been launched to resequence the genomes of at least a thousand people from around the world using the new sequencing technologies to produce the most detailed map of human genetic variation for disease studies. As the technologies mature and their uses spread, new sequencing-based methods to detect structural variations have been developed to take advantage of the massively parallel sequencing. In the read-depth approach, after DNA fragments are sequenced from one or both ends, the reads are mapped to the genome and then counted in a non-overlapping sliding window. Both methods provide a rapid, robust, and comprehensive approach to identify CNVs on the whole-genome scale.

Both array-CGH and read-depth sequencing generate genomic copy number (GCN) data in a very similar format: they consist of genomic signal output indexed by the genomic locations. The signals are log-ratios of normalized intensities from the test sample to those from the reference sample for array-CGH and sequence read counts after mean subtraction for read-depth sequencing, respectively. The goal of analyzing such data is to detect CNVs by identifying regions with signals that are consistently higher or lower than the normalized baseline. Implicitly, there are two distinct and yet closely related estimation problems: one is to estimate the number of CNVs, and the other is to determine the boundaries and the average signal strength of each of them. Many statistical and computational methods have been developed to identify CNVs in individual genomes. They include approaches built on hidden Markov model [11-13] or in a Bayesian framework [14-16]. Recently a method to identify recurrent CNVs within a group of individuals has also been proposed [17]. Based on their data analysis approaches, algorithms that have been developed to analyze such data can be roughly grouped into three types: some only smooth the raw log-ratio data and the regions with log-ratios higher or lower than a preset threshold are identified as CNVs [18,19], others estimate the number of CNVs and their boundaries directly using the original log-ratio data [20-23], and the rest use a combined approach [24-27]. The relative performance of these algorithms has been assessed [28].

Here we present a Bayesian statistical framework to analyze both array-CGH and read-depth data. Treating parameters that define the underlying genomic copy number variation encoded in the data as random variables, our approach derives the posterior distribution of those parameters given observed data. This statistical method models the location of regional changes and their corresponding associated copy number, and estimates the overall noise level in the data at the same time. Sampling from the posterior distribution using Markov chain Monte Carlo (MCMC) simulation is able to give both the best estimate and a corresponding Bayesian credible interval for each parameter in the model. We discuss how our model was derived and implemented, and the empirical results from applying our method to both microarray and sequencing data for CNV discovery.

## Statistical model

In the life sciences we are often faced with the task of making inferences about some object of interest given incomplete and noisy experimental data. In the case of CNV study, we are primarily concerned with inferring the number of the DNA copy number variations, their locations, sizes, and corresponding copy numbers-associated amplitude measurements, given the genomic copy number data, which are log-ratios of sample and control intensities measured on microarrays or read depths generated by shot-gun genomic sequencing. To demonstrate the application of our method to the read-depth data, we take a set of sequence reads from the 1000 Genomes Project and construct a 'read-depth intensity signal' spectrum by first mapping the reads to the human genome reference sequence and then counting the number of reads in a sliding window, a procedure that transforms sequencing data into array-like intensity signal. We capture these unknown quantities in a probability model that relates them to the observed data. Our model is Bayesian in essence as we assign prior distributions to parameters and use the posterior distribution function to estimate the underlying data generating process. Given the posterior distribution, we then use the Markov chain Monte Carlo method to fit the model. By doing so, we get not only the best estimates for these unknown parameters but also Bayesian credible intervals for the estimations at the same time.

Given a set of genomic copy number data ***D ***= {*g_k_*, *x_k_*}, *k *= 1, 2, ..., *M*, in which *g_k _*is the sorted genomic location of the *k*th data point, *x_k _*the signal at this location, and *M *the number of data points, we try to infer the genomic 'spectrum' ℱ, which is the unknown function defined by the CNVs encoded in the data set with the same measurement unit as *x_k_*. Assuming that the measurements of CNVs are all step functions, the spectrum ℱ can be written as

(1) $$f_{k}=\{\begin{array}{cc} a_{j} & \text{if}\;s_{j}\leq g_{k}<(s_{j}+w_{j}),j=1,2,\ldots ,N\\ 0 & \text{otherwise} \end{array},$$

where *N *is the number of 'smoothed' CNVs detectable in the data set, and *s_j_*, *w_j_*, *a_j _*are the start genomic location, the width, and the amplitude of the *j-*th CNV respectively. Thus the 'ideal' data set corresponding to ***D ***based on this model is ***F ***= {*g_k_*, *f_k_*}, *k *= 1, 2, ..., *M*. For simplicity, we assume that *X_1_*, *X_2_*, ..., *X_M _*measured in ***D ***are independent random variables each subjected to additive Gaussian noise around ℱ with a standard deviation *σ*.

**Figure 1:**
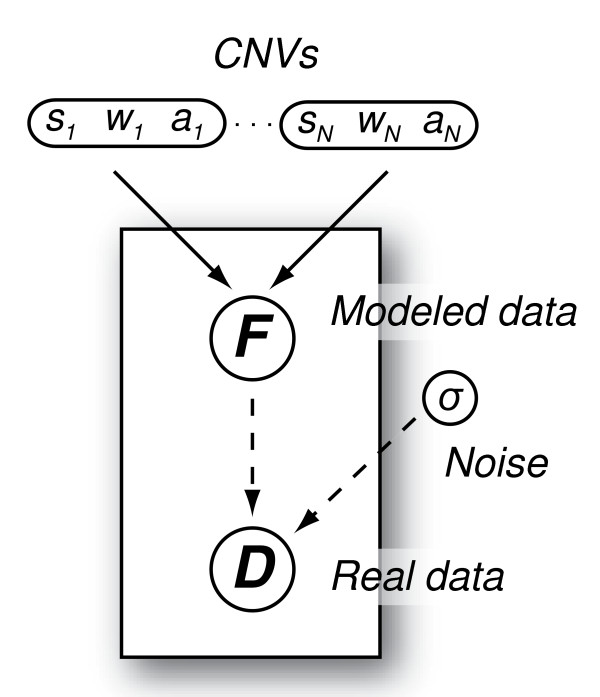
**The graphical representation of the proposed Bayesian model**. All quantities are shown as nodes in this directed graph. The parameter triplets *s_j_*, *w_j_*, and *a_j _*(*j *= 1, 2, ..., *N*) are combined together. The solid arrows indicate the modeled data set ***F ***is determined by a logical function with parameters *s_j_*, *w_j_*, and *a_j _*(*j *= 1, 2, ..., *N*), and the dashed arrows signify the stochastic dependence of the real data set ***D ***on both ***F ***and the overall data noise level *σ*.

Given the aforementioned model (Figure 1), the set of parameters to be inferred from ***D ***is ***θ ***= {*N*, (*s_j_*, *w_j_*, *a_j_*), *σ*^2^}, *j *= 1, 2, ..., *N*. Sometime for convenience, instead of reporting the estimate of *w_j_*, we report the estimate of *e_j_*, the end of the *j-*th CNV (*e_j _*= *s_j _*+ *w_j _- *1). The conditional probability distribution function *p*(***θ***|***D***) summarizes our inference about ***θ ***given the data and our prior knowledge about the CGH spectrum ℱ.

Bayes' theorem relates the posterior probability distribution function *p*(***θ***|***D***) to the likelihood probability distribution function *p*(***D***|***θ***) that can be calculated from the data and the prior probability distribution function *p*(***θ***) that encodes the prior knowledge,

(2) $$p(\theta |\text{D})=\frac{p(\text{D}|\theta )\cdot p(\theta )}{p(\text{D})}\propto p(\text{D}|\theta )\cdot p(\theta ),$$

where the normalization constant *p*(***D***) is omitted for simplicity.

Likelihood. Given the simplifying normality assumption stated above, the likelihood function takes the form

(3) $$p(\text{D}|\theta )=\frac{e^{-\psi^{2}/2}}{{\left(2Ũ\sigma^{2}\right)}^{M/2}},$$

where

(4) $$\psi^{2}=\sum_{k=1}^{M}\frac{{\left(x_{k}-f_{k}\right)}^{2}}{\sigma^{2}}.$$

Prior. Given the discrete nature of CNVs, it is reasonable to assume *a priori *independence among all the parameters in ***θ***. We choose the following prior distributions:

▫ Uniform distributions for *N*, *s_j_*, and *w_j _*(*j *= 1, 2, ..., *N*):

• *p*(*N*) = 1/*N*_max_

• *p*(*s_j_*) = 1/(*s*_max-_*s*_min_) = 1/*M*

• *p*(*w_j_*) = 1/*M*

▫ Normal distribution for *a_j_*: *p*(*a_j_*) ~ N(*τ_j_*, *κ_j_*^2^)

▫ Inverse gamma distribution for $\sigma^{\text{2}}:p\left(\sigma^{\text{2}}\right)=\beta^{\alpha }\sigma^{-\text{2}(\alpha +\text{1})}e^{-\beta /\sigma^{\text{2}}}/\Gamma \left(\alpha \right)$

Thus the prior probability distribution function is assigned as

$$p(\theta )=\frac{1}{N_{\max}}\cdot \frac{1}{M^{2N}}\cdot \frac{\beta^{\alpha }}{\Gamma (\alpha )}\sigma^{-2(\alpha +1)}e^{-\beta /\sigma^{2}}\cdot \prod_{j=1}^{N}\frac{1}{\sqrt{2Ũ\kappa_{j}^{2}}}e^{-{\left(a_{j},-\tau_{j}\right)}^{2}/2\kappa_{j}^{2}}.$$

After rearrangement and removal of the constant *N*_max_, we have

(5) $$p(\theta )\propto \frac{1}{M^{2N}\cdot \sigma^{2(\alpha +1)}\cdot {\left(2Ũ\right)}^{N/2}\cdot \prod_{j=1}^{N}\kappa_{j}\cdot e^{\beta /\sigma^{2}+\Sigma_{j=1}^{N}{\left(a_{j},-\tau_{j}\right)}^{2}/2\kappa_{j}^{2}}},$$

where *α*, *β*, *τ_j _*and *κ_j _*are the hyperparameters that characterize the prior distribution. See the Implementation subsection below for their parameterization.

Posterior. Substituting the product of the likelihood and the prior of equations (3) and (5) into equation (2), we obtain

(6) $$p(\theta |D)\propto \frac{1}{M^{2N}\cdot \sigma^{2(\alpha +1)+M}\cdot {\left(2Ũ\right)}^{N/2}\cdot \prod_{j=1}^{N}\kappa_{j}\cdot e^{\psi^{2}/2+\beta /\sigma^{2}+\Sigma_{j=1}^{N}{\left(a_{j},-\tau_{j}\right)}^{2}/2\kappa_{j}^{2}}}.$$

For a given model {ℳ: ***θ ***∈ Θ}, where *N *is known and thus ***θ ***= {(*s_j_*, *w_j_*, *a_j_*), *σ*^2^}, *j *= 1, 2, ..., *N*, the posterior distribution of ***θ ***given the data ***D ***and the model ℳ can be expressed as

(7) $$p(\theta |\text{D})\propto \frac{1}{\sigma^{2(\alpha +1)+M}\cdot e^{\psi^{2}/2+\beta /\sigma^{2}+\Sigma_{j=1}^{N}{\left(a_{j},-\tau_{j}\right)}^{2}/2\kappa_{j}^{2}}}.$$

Informative prior. If we have information on certain parameters in ***θ***, for example *s_j _*and *w_j_*, from an initial scan of data ***D***, such information can be coded in an informative prior to simplify subsequent parameter estimation. For example, suppose we know *N *= 1, the CNV starts at a certain place between genomic position *a *and *b*, and its length is between *c *and *d *bp long. We code such prior information as following:

▫ Uniform distributions for *s*_1 _and *w*_1_:

• $\;p(s_{1})=\{\begin{array}{c} \frac{1}{b-a}\text{for}\;s_{1}\in [a,b]\\ 0\text{otherwise} \end{array}$

• $\;p(w_{1})=\{\begin{array}{c} \frac{1}{d-c}\text{for}\;w_{1}\in [c,d]\\ 0\text{otherwise} \end{array}$

Keeping priors on other parameters the same as before, we have

(8) $$p(\theta )\{\begin{array}{cc} \propto \frac{1}{\sigma^{2(\alpha +1)+M}\cdot e^{\psi^{2}/2+\beta /\sigma^{2}+{\left(a_{1}-\tau_{1}\right)}^{2}/2\kappa_{1}^{2}}} & \text{for}\;s_{1}\in [a,b]\;\text{and }w_{1}\in [c,d]\\ =0 & \text{otherwise}\;\;\;\;\;\;\;\;\;\;\;\;\;\;\;\;\;\;\;\;\;\;\;\; \end{array}.$$

With this informative prior, the posterior is the same as (7), but only none-zero for *s*_1 _∈[*a*, *b*] and *w*_1 _∈ [*c*, *d *]. This condition simplifies subsequent parameter estimation, as *s*_1 _and *w*_1 _only need to be sampled in these two intervals during MCMC simulation.

In some case, we only know the start and the length of a particular CNV (similar to the case above) but still have to estimate *N *and the parameters of the other CNVs. This is a case that mixes the general and the special ones presented above. It is easy to show the informative prior is a mix of (7) and (8):

$$p(\theta )\{\begin{array}{cc} \propto \frac{1}{M^{2N}\cdot \sigma^{2(\alpha +1)}\cdot {\left(2Ũ\right)}^{N/2}\cdot \prod_{j=1}^{N}\kappa_{j}\cdot e^{\beta /\sigma^{2}+\Sigma_{j=1}^{N}{\left(\alpha_{j}-\tau_{j}\right)}^{2}/2\kappa_{j}^{2}}} & \text{for}\;s_{1}\in [a,b]\;\;\text{and}\;w_{1}\in [c,d]\\ \;=0 & \text{otherwise}\;\;\;\;\;\;\;\;\;\;\;\;\;\;\;\;\;\;\;\;\;\;\;\;\;\;\;\;\;\;\;\;\;\;\;\;\; \end{array}\;.$$

When analyzing clean read-depth data, if the amplitude of the *j-*th CNV, *a_j_*, occurs discretely at several different values (for example *a_j _*∈ {-c, c, 2c}, where c is the genome-wide average haploid read depth), the prior distribution *p*(*a_j_*) of *a_j _*can be modeled naturally by a multinomial distribution.

## Algorithm and implementation

### Parameter estimation by Markov chain Monte Carlo simulation

Analytically summarizing the posterior distribution *p*(***θ***|***D***) is difficult. For example, even though in theory the posterior expectation of an arbitrary function of ***θ***, *g*(***θ***), can be computed as

(9) $$E(g(\theta )|D)=\int_{\theta }g(\theta )p(\theta |D)d\theta ,$$

the calculation is usually impracticable for two reasons. Firstly, *p*(***θ***|***D***) is only known up to some multiplicative constant due to the proportionality form of equation (8). Secondly, even if the exact form of *p*(***θ***|***D***) is known, given the number of parameters in ***θ ***(at least four in a non-trivial case), the high dimensional integral required in equation (8) is very difficult to be carried out in practice and soon becomes intractable as the number of parameters increases. However, Markov chain Monte Carlo (MCMC) provides an alternative whereby the posterior can be directly sampled to obtain sample estimates of the quantities of interest. Thus using a random sequence of ***K ***draws ***θ***^(1)^, ***θ***^(2)^, ..., ***θ***^(*K*) ^from *p*(***θ***|***D***), *E*(*g*(***θ***)|***D***) can be approximated by simply taking the average of these draws. Similar methods can be used to compute the posterior standard deviation $\zeta_{\hat{\theta }}$ or quantiles, probabilities that parameters take particular values, and other quantities of interest.

The Gibbs sampling algorithm [29] was implemented to sample from the target distribution {*p*(***θ***|***D***,ℳ): ***θ ***∈ Θ ⊆ ℝ^3*N*+1^}. To do so, the Gibbs sampler first constructs an aperiodic and irreducible Markov chain whose stationary distribution is *p*(***θ***|***D***,ℳ) in the state space Θ, and then draws a sequence of random samples from conditional distributions to characterize the joint target distribution. More precisely, it was implemented by (i) taking some initial values ***θ***^(0)^; (ii) repeating for each *t *= 1, 2, ..., *T*, where *T *is the preset number of iterations, generating ***θ***_*i*_^(*t*) ^from *p*(***θ***_*i*_^(*t*)^| ***θ***_1_^(*t*)^, ..., ***θ***_*i*-1_^(*t*)^, ***θ***_*i*+1_^(*t*-1)^, ..., ***θ _||θ||_***^(*t*-1)^,***D***,ℳ) for *i *= 1, 2, ..., ***||θ||***; (iii) continuing the previous step for *T *times after the estimated target distribution $\hat{p}(\theta |D,ℳ)$ converges.

To calculate the conditional probabilities of *s_j _*and *w_j _*required by the second step of the Gibbs sampling stated above, all possible *s *∈ [*g*_1_, *g_M_*] and *w *∈ [*w*_min_, *w*_max_] are evaluated. Given the normality assumption about the data, conjugate prior distributions of *a_j _*and ***σ***^2 ^can be used to simplify the calculation of their conditional probabilities. If the prior distribution of *a_j _*takes the conjugate from *p*(*a_j_*) ~ N(*τ_j_*, *κ_j_*^2^), the conditional distribution of *a_j _*given other parameters, the data ***D***, and the model ℳ is also a normal distribution as $N([(1/\kappa_{j}^{2})/(1/\kappa_{j}^{2}+w_{j}/\sigma^{2})]\;\tau_{j}+[(w_{j}/\sigma^{2})/(1/\kappa_{j}^{2}+w_{j}/\sigma^{2})]\;{\bar{x}}_{j}$

, $1/(1/\kappa_{j}^{2}+w_{j}/\sigma^{2}))$ where ${\bar{x}}_{j}$ is the average log-ratios of probe intensities in the *j-*th CNV. Given the conjugate prior distribution of *σ*^2^, *p*(*σ*^2^) ~ ℐnvmma(*α*, *β*), the conditional distribution of *σ*^2 ^given other parameters, the data ***D***, and the model ℳ is also an inverse gamma distribution, $ℐ\text{nv}G\text{amma}(\alpha +M/2,\;\beta +\sum_{i=1}^{M}{\left(x_{j}-\bar{x}\right)}^{2}/2)$.

### Model selection using Bayes factor

Model selection is required to determine *N*, the number of CNVs, as different *N *changes the model parameterization ***θ***. Suppose that the data ***D ***have arisen under one of the two models, {ℳ_1_: ***θ***_1 _∈ Θ_1_} and {ℳ_2_: ***θ***_2 _∈ Θ_2_}, according to a probability density *p*(***D ***|ℳ_1_) or *p*(***D ***|ℳ_2_).

Given prior probabilities *p*(ℳ_1_) and *p*(ℳ_2_) = 1 - *p*(ℳ_1_), the data produce posterior probabilities *p*(ℳ_1_|***D***) and *p*(ℳ_2_|***D***) = 1- *p*(ℳ_1_|***D***). From Bayes' theorem, we obtain

$$p({ℳ}_{j}|\text{D})=\frac{p({ℳ}_{j})p(\text{D}|{ℳ}_{j})}{p({ℳ}_{1})p(\text{D}|{ℳ}_{1})+p({ℳ}_{2})p(\text{D}|{ℳ}_{2})}(j=1,2),$$

so that

(10) $$\frac{p({ℳ}_{1}|\text{D})}{p({ℳ}_{2}|\text{D})}=\frac{p({ℳ}_{1})}{p({ℳ}_{2})}\cdot \frac{p(\text{D}|{ℳ}_{1})}{p(\text{D}|{ℳ}_{2})}.$$

For a given model {ℳ: ***θ ***∈ Θ}, *p*(***D ***|ℳ) can be approximated by the sample harmonic mean likelihoods,

(11) $${\hat{p}}_{\text{HM}}(D|ℳ)={\left[\frac{1}{K}\sum_{i=1}^{K}\frac{1}{p(D|\theta^{\left(i\right)},ℳ)}\right]}^{-1},$$

based on *K *MCMC draws ***θ***^(1)^, ***θ***^(2)^, ..., ***θ***^(*K*) ^from the posterior distribution *p*(***θ***|***D***). The harmonic mean estimator is consistent since ${\hat{p}}_{\text{HM}}(\text{D}|ℳ)\to p(D|ℳ)$ as *K *→ + **∞**. It may, however, have infinite variance across simulations. To solve this problem, Newton and Raftery [30] proposed an alternative estimator,

(12) $${\hat{p}}_{4}(D|ℳ)=\frac{m\delta /(1-\delta )+\sum_{i=1}^{K}p(D|\theta^{\left(i\right)},ℳ)/\left[\delta {\hat{p}}_{4}(\text{D}|ℳ)+(1-\delta )p\left(D|\theta^{\left(i\right)},ℳ\right)\right]}{m\delta /(1-\delta ){\hat{p}}_{4}(\text{D}|ℳ)+{\sum_{i=1}^{K}\left[\delta {\hat{p}}_{4}(\text{D}|ℳ)+(1-\delta )p(D|\theta^{\left(i\right)},ℳ)\right]}^{-1}},$$

which does not display any of the instability of ${\hat{p}}_{\text{HM}}(D|ℳ)$. We implemented ${\hat{p}}_{4}(D|ℳ)$ to calculate the Bayes factor for model comparison

### Implementation

Our method, including the Markov chain Monte Carlo simulation and the model comparison, is currently implemented in R [31]. To use non-informative priors for our Bayesian inference, we set *τ_j _*= 0 and *κ_j _*= 100, which effectively makes the prior distribution of *a_j _*flat around 0. We also assign 1 to both *α *and *β *for the inverse gamma distribution of *σ*^2^. We tested various values of the hyperparameters (*τ_j_*, *κ_j_*, *α*, and *β*), and the simulation results showed that the parameter inference was insensitive to the values assigned to these hyperparameters, which is expected given the large number of data points. A 500-iteration MCMC simulation of the posterior distribution (7) given a data set with *M *= 500 and *N *= 1 took 126 seconds of CPU time on a personal computer with one 1400-Mhz x86 Intel processor and 500 MB RAM. To assess the convergence of the Markov chain after 500 iterations, we started multiple chains from different values of ***θ***^(0)^. The simulations showed that after initial dozens of iterations all chains converged to the same solution. Based on this observation, we concluded that 500 iterations were sufficient for Bayesian inference in this case. We used the same convergence diagnostic for all inferences.

Because of great computational intensity of the MCMC simulation, to process a large GCN data set, we use a 'divide and conquer' strategy. We first sort array/sequencing features from each chromosome according to their genomic locations and then group 1000 consecutive features into subsets for parallel processing on a computer cluster.

## Results

### Simulated array-CGH data

**Figure 2:**
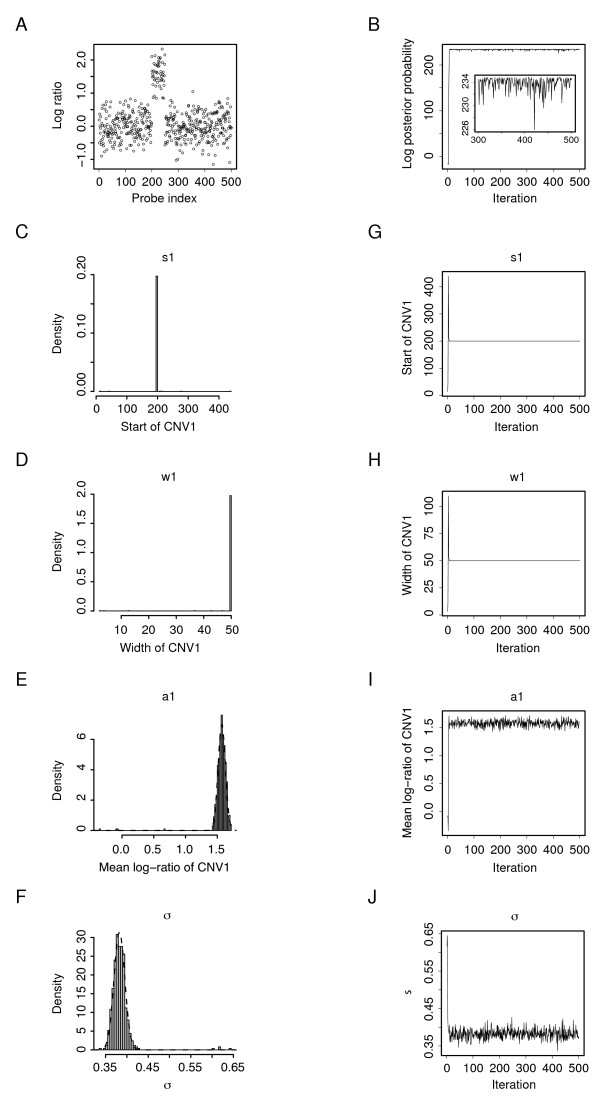
**Parameter estimation by MCMC simulation for a simulated array-CGH data set**. (A) The log-ratio vs. probe genomic index plot of a simulated one-CNV array-CGH data set. The data ***D ***(*M *= 500) were generated with ***θ ***= {*N *= 1, (*s*_1 _= 200, *w*_1 _= 50, *a*_1 _= 1.5), *σ*^2 ^= 0.4^2^}. (B) The logarithm of the posterior probability (calculated up to some multiplicative constant) at consecutive 500 MCMC sampling iterations. In the stationary phase, the posterior probability of the MCMC-sampled parameter values given data ***D***, $p(\hat{\theta }|D)$, fluctuates closely beneath the maximum value *p*(***θ***|***D***). (C-F) Histograms of the 500 estimates of *s*_1_, *w*_1_, *a*_1_, and *σ *respectively. (G-J) Traces of the estimates of *s*_1_, *w*_1_, *a*_1_, and *σ *through the consecutive 500 MCMC sampling iterations.

**Figure 3:**
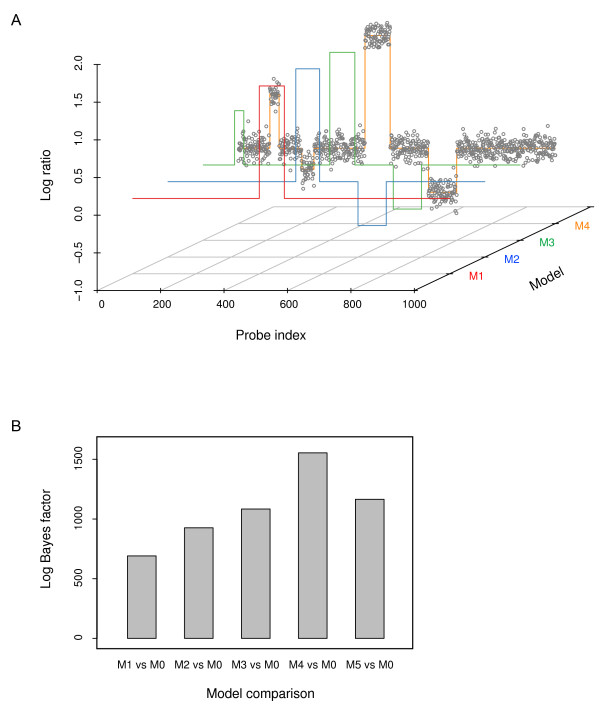
**Model selection**. (A) A simulated four-CNV array-CGH data set. The data D (M = 1000) were generated with ***θ ***= {*N *= 4, (*s*_1 _= 100, *w*_1 _= 30, *a*_1 _= 0.7), (*s*_2 _= 200, *w*_2 _= 20, *a*_2 _= -0.3), (*s*_3 _= 400, *w*_3 _= 80, *a*_3 _= 1.5), (*s*_4 _= 600, *w*_4 _= 90, *a*_4 _= -0.6), *σ*^2 ^= 0.1^2^}. The scatter plot of the multi-CNV array-CGH data are overlaid with the segmentation found by our algorithm using different models. The step function lines in red, green, blue, and orange represent models with *N *= 1, 2, 3, and 4, respectively. For figure clarity, the estimated model with *N *= 5 is not shown. (B) Model comparison. The comparison between each of the models with *N *= 1, 2, 3, 4 and the basal, null model (*N *= 0) was quantified by the logarithm of the Bayes factor.

We first used simulated array-CGH data sets to test our Bayesian model and its implementation. To generate such synthetic data, we first specified values for the parameters in ***θ ***= {*N*, (*s_j_*, *w_j_*, *a_j_*), *σ*^2^}, *j *= 1, 2, ..., *N*, in which *N *and (*s_j_*, *w_j_*, *a_j_*) define the artificial genomic CNV structure encoded as a step function and *σ*^2 ^determines the overall noise level in the data. The simulated data were then generated by superimposing this predefined step function with random Gaussian noise. Typical simulated array data with one and multiple CNVs are shown in Figures 2A and 3A respectively.

The simulated array data (*M *= 500) plotted in Figure 2A were generated with ***θ ***= {*N *= 1, (*s*_1 _= 200, *w*_1 _= 50, *a*_1 _= 1.5), *σ*^2 ^= 0.4^2^}, which was to be estimated. Taking *N *= 1, we started the Markov chain at some random ***θ***^(0) ^= {(*s*_1 _= 100, *w*_1 _= 0, *a*_1 _= 0), *σ*^2 ^= 0.1^2^} and ran it for 500 iterations. The sampling results are shown in Figure 2C-F. As the parameter trace plots (Figure 2G-J) show, the Markov chain quickly converged to stationarity after approximately ten iterations (Figure 2B). To err on the side of caution, we discarded the samples from the first 100 iterations as the 'burn-in' samples and estimated the parameter values from the rest 400 samples, which gave $\hat{\theta }=\{({\hat{s}}_{1}=200,{\hat{w}}_{1}=50,{\hat{a}}_{1}=1.57),{\hat{\sigma }}^{2}=0.38^{2}\}$ given *N *= 1.

Remarkably, all these samples have the very similar *s*_1 _and *w*_1_, which are 200 and 50 respectively. Because of this small variation in their estimation, the estimates of *s*_1 _and *w*_1 _from the data are of extremely high confidence. The distributions of *a*_1 _and *σ *in the 400 samples are approximately normal as N(1.57, 0.057^2^) and N(0.38, 0.012^2^) respectively. Based on their normal distributions, we can easily calculate a Bayesian credible interval for both *a*_1 _and *σ*. For example, a 95% Bayesian credible interval for *a*_1 _is [1.46, 1.68], which suggests that, after observing the data, there is a 95% chance that the average log-ratio of intensities in this CNV falls between 1.46 and 1.68.

We also simulated array-CGH data (*M *= 1000) with multiple CNVs (Figure 3A) using ***θ ***= {*N *= 4, (*s*_1 _= 100, *w*_1 _= 30, *a*_1 _= 0.7), (*s*_2 _= 200, *w*_2 _= 20, *a*_2 _= -0.3), (*s*_3 _= 400, *w*_3 _= 80, *a*_3 _= 1.5), (*s*_4 _= 600, *w*_4 _= 90, *a*_4 _= -0.6), *σ*^2 ^= 0.1^2^}. To identify the CNVs encoded in this data set, first the model-specific parameters {(*s_j_*, *w_j_*, *a_j_*), *σ*^2^}, *j *= 1, 2, ..., *N *were estimated under different models with *N *= 0, 1, ..., 5. In Figure 3A, the scatter plot of the multi-CNV array-CGH data are overlaid with the segmentation found by our algorithm using different models. The figure shows that the most prominent CNV was identified first when the number of CNVs, *N*, was set to 1 and less prominent CNVs were progressively identified as the model became more permissive (i.e., *N *was increased). To select the most plausible model from which the observed data were generated, each of the models with *N *= 1, 2, 3, 4, 5 was then compared with the basal, null model (*N *= 0). Quantification of these comparisons by the logarithm of the Bayes factor, which gives 691.02, 926.94, 1091.13, 1556.23, and 1173.67 respectively, clearly indicates that the model with *N *= 4 is the best model among the ones tested (Figure 3B). It is noteworthy that since the numbers aforementioned are the logarithms of the Bayes factor the actual increase in the marginal evidence *p*(***D***|M) between neighboring models is very substantial. For example, the increase in the marginal evidence from *N *= 3 to *N *= 4 is *e*^1556.23-1091.13 ^= *e*^465.11 ^≈ 9.86 × 10^201 ^fold.

**Figure 4:**
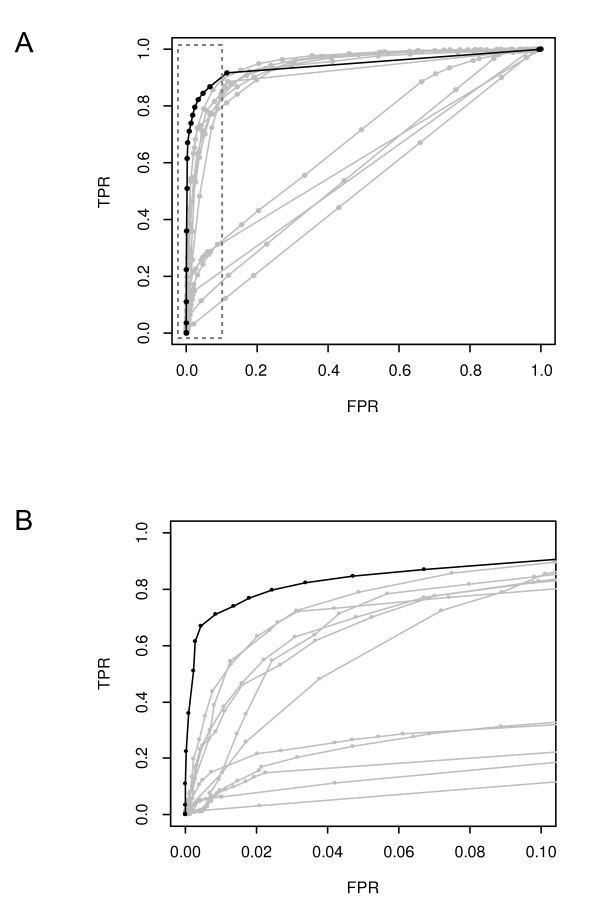
**The ROC curves of array-CGH data analysis methods**. (A) The complete plot. All curves were calculated using the same data set. They show that our Bayesian algorithm (black line) is appreciably more sensitive than all other methods (gray lines) at low (< 10%) false positive rates. (B) Details of the ROC curves in the low FPR region of (A) inside the box with dashed border. See Lai et al. [28] for the identities of the gray ROC curves. TPR, the true positive rate; FPR, the false positive rate.

Lai et al. [28] examined the performance of 11 array-CGH data analysis methods: CGHseg, quantreg, CLAC, GLAD, CBS, HMM, wavelet, lowess, ChARM, GA, and ACE. To assess the performance of our algorithm in conjunction with these methods, we used the same simulated data as Lai et al. used for the assessment in their study to calculate the true positive rates (TPR) and the false positive rates (FPR) as the threshold for determining a CNV is varied. See Lai at al. for the definitions of TPR and FPR and the details of the simulated data sets. We calculated the receiver operation characteristic (ROC) curve of our algorithm using the most noisy (thus the lowest signal-to-noise ratio, SNR = 1) data set with the CNV width of 40 probes. This ROC curve, together with the ROC curves of other array-CGH methods based on the same data set, was plotted in Figure 4. These curves show that our Bayesian algorithm is appreciably more sensitive than all other methods at low (< 10%) false positive rates. We need to point out that the comparison was conducted in a fair manner, if not to the disadvantage of our method: all the results from Lai et al. were used directly without modification and our method has no free parameters to tune.

### Glioblastoma Multiforme array-CGH data

Lai et al. [28] compared 11 different array-CGH data analysis algorithms that are based on diverse statistical or computational techniques. In addition to testing those methods using simulated data, they also characterized their performance on chromosomal regions of interest in real data sets obtained from patients with Glioblastoma Multiforme (GBM) [32]. These cDNA microarray-based data sets were generated by CGH-profiling copy number alterations across 42,000 mapped human cDNA clones, in a series of 54 gliomas of varying histogenesis and tumor grade.

**Figure 5:**
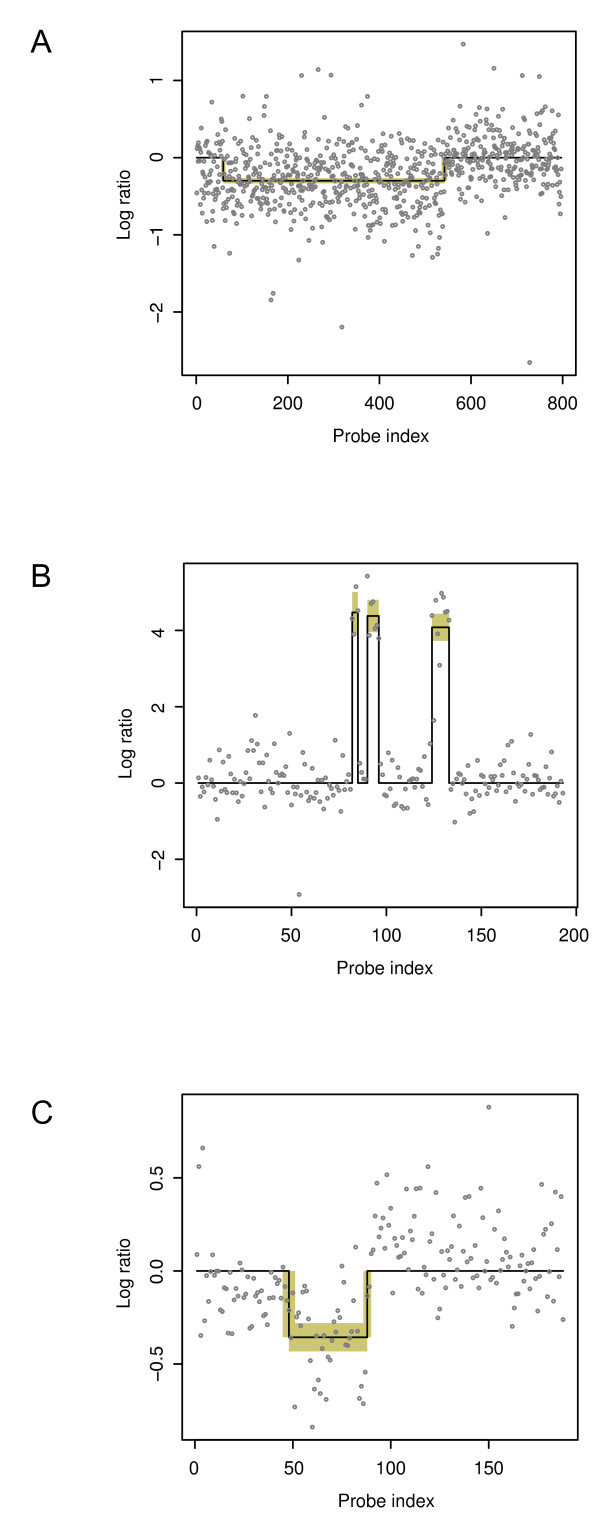
**CNV profiles of three experimental array-CGH data sets**. Probe segmentation of (A) GBM31, (B) GBM29, and (C) *β*-globin small deletion array-CGH data. In all cases, the MCMC sampler was run for 1000 iterations. The first 100 samples were discarded as the 'burn-in' samples, and the mean and the standard deviation of each parameter were estimated from the rest 900 samples. The estimated means are plotted as a step function (the black line), and the estimated standard deviations are indicated as yellow boxes, each defined by mean ± 1.96 × standard deviation, which corresponds to a 95% credible interval.

It was observed that the GBM data contain a mixture of larger CNV regions with low amplitude and smaller ones with high amplitude. These two types of array-CGH data are nicely represented by data sets GBM31 and GBM29 respectively (Figure 5A-B). In sample GBM31, a large region on chromosome 13 was lost, and the overall magnitude of this loss is very low due to the low penetrance of this genetic variation in tumor cells in this sample. In sample GBM29, on the other hand, there are three high-amplitude small duplications. To evaluate our Bayesian approach in a comparable way, we also used these two GBM data sets processed and utilized by Lai et al. to test our method.

Figures 5A and 5B show the array-CGH profiles of chromosomes 13 and 22 in Glioblastoma Multiforme samples GBM31 and GBM29, respectively, overlaid with the segmentation found by our algorithm. As seen in Figure 5A, our algorithm detected the single broad proximal deletion of part of chromosome 13 in GBM31, spanning from the 59^th ^to the 542^nd ^probe with a log-ration intensity at -0.30 $\left(\;\;\hat{\theta }=\{(\;{\hat{s}}_{1}=59,\;\;\;{\hat{e}}_{1}=542,\;\;{\hat{a}}_{1}=-0.30),\;\;\;{\hat{\sigma }}^{2}=0.38^{2}\right\}$ with corresponding standard deviations, $\zeta_{\hat{\theta }}=\{(\zeta_{{\hat{s}}_{1}}=2.40,\;\zeta_{{\hat{e}}_{1}}=3.19,\;\zeta_{{\hat{a}}_{1}}=-0.02),\;\zeta_{{\hat{\sigma }}^{2}}=0.01\}$, for calculating each Bayesian credible interval). The breakpoint ${\hat{e}}_{1}$ at the probe genomic index 542 was also identified by all the programs that detected this deletion in the test conducted by Lai et al. The other breakpoint ${\hat{a}}_{1}$ at 59 was again found by CLAC and ACE evaluated in the same test [28]. The small sample standard deviations in $\zeta_{\hat{\theta }}$ connote the reliability of the parameter estimation despite a rather low signal to noise ratio of the GBM31 data. Our algorithm also detected all three high-amplitude amplifications of parts of chromosome 22 in GBM29 (Figure 5B). Even though there are only four probes separating the first two amplifications, our method still segmented them clearly. Moreover, our method also pinpointed the six breakpoints of these three CNVs (their sample standard deviations are all zeros), which makes these predictions highly reliable.

### β-globin high-density array-CGH data

One recent significant development in the microarray technology is the emergence of the tiling array technology, which can be used to cover large genomic regions or even an entire genome on one or several microarrays in an unbiased fashion by using oligonucleotides (a.k.a. tiles) uniformly sampled from presented genomic sequences. The current trend is to migrate from PCR-based arrays to tiling arrays for a much higher resolution and a comprehensive genomic coverage.

In a recent study [7], in order to test the resolution limits of tiling arrays when they are used with CGH for CNV discovery, Urban et al. designed microarrays that tile through 100 kb of the *β*-globin locus with overlapping isothermal oligonucleotides spaced 9 bp apart alone the tiling path. They compared the test DNA from a patient with a known heterozygous deletion of 622 bp in the *β*-globin locus and the reference DNA pooled from seven individuals without this chromosomal aberration. Figure 5C shows the array-CGH profile of the *β*-globin locus of the patient overlaid with the segmentation $\left(\;\hat{\theta }=\{(\hat{s}=48,\hat{e}=88,\hat{a}=-0.36),\;\;{\hat{\sigma }}^{2}=0.25^{2}\}\right)$ found by our algorithm. This deletion in the *β*-globin locus was detected, and the estimate of its length, $\hat{w}$, corresponding to 641 bp in the genomic coordinate system, is highly accurate in comparison with the actual length of the deletion (622 bp).

### Read-depth genome resequencing data

The genome of a Utah resident with Northern and Western European ancestry from the CEPH collection (NA12878) has been sequenced by the 1000 Genomes Project using both the 454 paired-end and the Illumina shot-gun sequencing technologies, which produced long (120-bp) sequence reads with low coverage (0.5×) and short (50-bp) ones with high coverage (40×), respectively.

**Figure 6:**
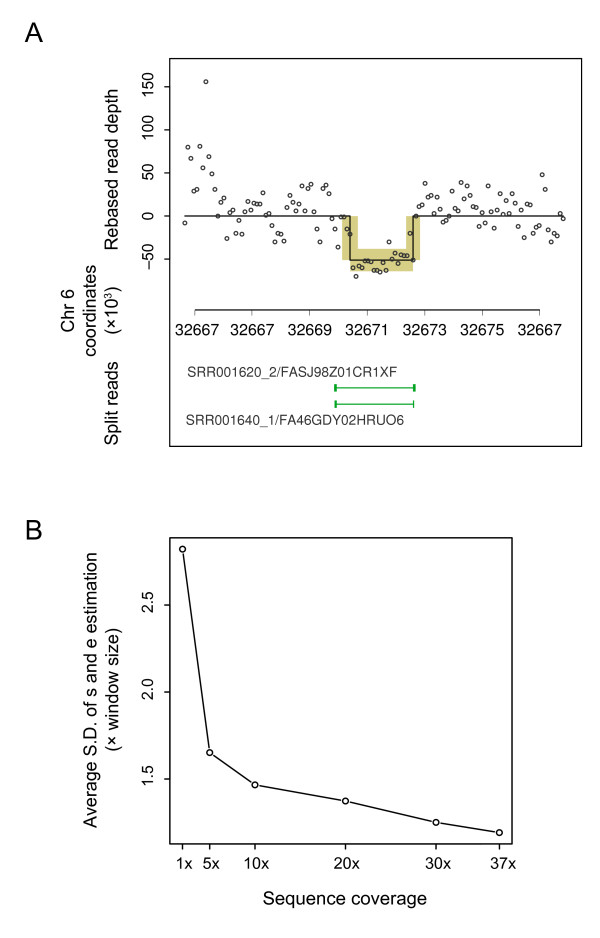
**Identification of CNV in the read-depth data**. (A) CNV profile of a deletion locus in a read-depth data set. Short reads generated for a CEPH individual were mapped to the human reference genome and then counted in a 100-bp non-overlapping sliding window to produce the read-depth data. The counts are centered to their mean. The MCMC sampler was set up in a similar fashion as in the previous cases. The estimated means are plotted as a step function, and the 95% credible intervals as yellow boxes. The 2653-bp deletion, which is found by long sequence reads that encompass this deletion locus and thus split around it when aligned to the reference sequence, is taken as known and shown as the thin green line on a lower track. (B) The averaged standard deviation in the estimates of the start and the end positions (*s *and *e*) at different sequencing depths. The S.D. unit is the window size, which is 200 bp in this case.

After using these two sequence sets to generate the 'known' genomic deletions in and the read-depth data from this individual, we apply our method to the read-depth data and compare the finding with the 'known' genomic deletions. Despite a very low sequencing depth, we are able to use 454 reads to detect several large genomic deletions in this individual based on the gapped (i.e., 'split') alignment of some of these long reads. These deletions are taken as known, and we use a 2653-bp deletion on chromosome 6 from 32,669,938 to 32,672,591 to illustrate the application of our read-depth method. After mapping approximately 2.4-billion 50-bp Illumina reads to the human reference genome, we count the number of reads in a 200-bp non-overlapping sliding window to produce the read-depth data. Figure 6A shows the read distribution profile based on the Illumina short reads surrounding the 2653-bp deletion locus.

Our method detected this deletion in the read-depth data and estimated its parameters to be $\hat{\theta }=\{(\;\;\hat{s}=32670400,\;\;\;\hat{e}=32672500,\;\;\hat{a}=-51.20),\;\;{\hat{\sigma }}^{2}=27.73^{2}\}$. To investigate how the sequencing depth affects the estimation of the start and the end positions of a CNV, we simulate a series of sequencing depths by randomly sampling (without replacement) different numbers of mapped Illumina reads and then apply our method to the simulated data. The standard deviation in the estimates of the start and the end positions, *s *and *e*, reflects how well these two parameters can be estimated from the read-depth data. In figure 6B we plot the averaged standard deviation in the estimates of the *s *and *e *at different sequencing depths. It is clear as the sequencing depth decreases from the original depth (37×) the estimates of the terminal positions become less accurate. In fact, when the coverage is below 1×, it becomes very difficult to find the deletion at all.

## Discussion and Conclusion

The Metropolis-Hastings and the Gibbs sampling algorithms, two Markov chain Monte Carlo simulation methods, have been widely used for Bayesian inference. Developed by Metropolis et al. [33] and subsequently generalized by Hastings [34], the Metropolis-Hastings algorithm generates, based on the current state and a pre-selected proposal density, candidate states that are accepted (or rejected) stochastically with a certain acceptance probability but then retains the current value when rejection takes place. Gibbs sampling [29] draws a sequence of random samples from conditional distributions of unknown parameters to characterize their joint target distribution. In fact, the Gibbs sampling can be regarded as a special case of the Metropolis-Hastings algorithm as the acceptance probability is always one--i.e., every proposal is automatically accepted.

For our Bayesian analysis of genomic copy number data, we implemented both the random walk Metropolis-Hastings (RWMH) and the Gibbs sampling algorithms and observed that in this application Gibbs sampling is much more suitable for parameter inference. RWMH worked well for one-CNV data. However, if the data contain two widely separated CNVs, it can only identify one of them but not both. To investigate this limitation, we plotted the landscape of the posterior probability distribution in a two-dimensional parameter space. A two-CNV data set ***D ***(*M *= 700) with ***θ ***= {*N *= 2, (*s*_1 _= 100, *w*_1 _= 20, *a*_1 _= 2), (*s*_2 _= 600, *w*_2 _= 20, *a*_2 _= 2), *σ*^2 ^= 0.4^2^} was first simulated, and then the posterior probability was evaluated with various combinations of *s*_1 _and *s*_2 _while all other parameters were kept fixed at their true values.

**Figure 7:**
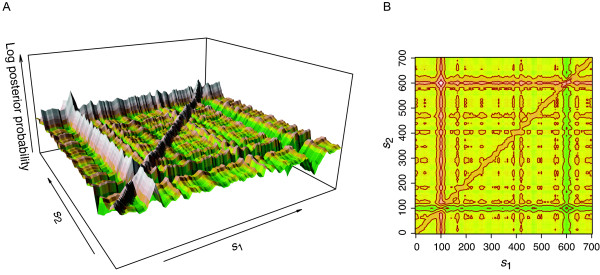
**The posterior distribution of model parameters**. (A) A view of the posterior probability distribution landscape in the two-dimensional *s_1_*-*s_2 _*parameter space. The simulated data ***D ***(*M *= 700) were generated with ***θ ***= {*N *= 2, (*s*_1 _= 100, *w*_1 _= 20, *a*_1 _= 2), (*s*_2 _= 600, *w*_2 _= 20, *a*_2 _= - 2), *σ*^2 ^= 0.4^2^}. The posterior probability was evaluated with various combinations of s1 ands2 while all other parameters were kept fixed at their true values. The terrain color varies from green to yellow to red and then to gray as the posterior probability increases. (B) The contour of the same posterior distribution. A closed contour line traces points (*s*_1_, *s*_2_) of equal posterior probability density. As expected, the global maximum of the posterior probability occurs where *s*_1 _= 100 and *s*_2 _= 600, two values that were used to generate the underlying data.

The surface plot in Figure 7A shows a global maximum peak located at *s*_1 _= 100 and *s*_2 _= 600 as expected and an overall very rugged posterior distribution 'terrain': the landscape is full of local maxima with, especially, two prominent 'ridges' of local maxima at *s*_1 _= 100 and *s*_2 _= 600, respectively. It is clear from Figures 7A and 7B that if the Markov chain of RWMH gets to a local maximum on the ridge at *s*_1 _= 100 or *s*_2 _= 600 but fortuitously far from the global maximum, it will be trapped on the ridge and practically cannot reach the global peak if the random update interval is small (which is almost always the case). Based on these observations, we chose the Gibbs sampling algorithm for our Bayesian analysis of the genomic copy number data as the Gibbs sampler is well suitable to explore this 'ridged' terrain by using full conditionals to scan the landscape along ridges to find the global maximum.

As the ROC curves in Figure 4 show, our Bayesian algorithm is the most sensitive method at low (< 10%) false positive rates. This means that at a given low FPR our method can identify more true positive probes inside CNVs than other methods. When the FPR is higher, it is less sensitive than several methods, most of which find CNVs through data smoothing. However, this is hardly a disadvantage, as at high false positive rates the list of identified CNVs is awash with false positives, rendering the whole list practically unusable.

In addition to the improved sensitivity, our method also has several distinct advantages innate to its Bayesian approach. The confidence on an estimated parameter value can be assessed through its Bayesian credible interval. Akin to a confidence interval but with an intuitive probabilistic interpretation, a Bayesian credible interval is a range of values that contains the true parameter value with a certain probability. Through stochastic simulation, it is straightforward to summarize the otherwise analytically intractable joint posterior distribution of the unknown parameters and compute both the best estimate and a corresponding Bayesian credible interval for each parameter in the model. The availability of the intervals for *s_j_*, *e_j_*, and *a_j_*--the start and the end genomic locations and the copy number of each CNV--is unique to our Bayesian method, and these credible intervals can be especially useful.

Recent years have seen fast development of methodologies in different frameworks to detect CNVs in array-CGH data. For example, to detect CNV breakpoints, Shah et al. used a modified hidden Markov model (HMM) that is robust to outlier probes [13], while Rueda and D'az-Uriarte used a nonhomogeneous HMM fitted via reversible jump MCMC [12]. Pique-Regi et al. used piecewise constant (PWC) vectors to represent genome copy numbers and sparse Bayesian learning to detect CNV breakpoints [16]. Other methods for segmenting array-CGH data have also been implemented, including using Bayesian change point analysis [15], a spatially correlated mixture model [14], a Bayes regression model [35], and wavelet decomposition and thresholding [36].

Due to the computational intensiveness of its MCMC simulation, the method that we present here can be most advantageously used to refine CNVs detected by fast point-estimate methods. It could also be seen as a basic genomic copy number data analysis framework, amenable for several possible extensions. Firstly, due to the nature of the genomic sequence duplications and deletions, the signal measurements of CNVs will aggregate to certain expected values. Such information could be incorporated into the model for better signal detection from background noise. Secondly, more complicated likelihood function, such as a truncated Gaussian, could be used to handle outliers in genomic copy number data. Thirdly, informative priors could be used for better CNV detection. The formation of CNVs in a genome is potentially affected by many local genomic features, such as conservation and repeat content on the sequence level. Compared with the aforementioned methods for array-CGH data, our Bayesian approach has the advantage to readily incorporate such sequence information through the prior distributions, as it treats the start and the width of CNVs as parameters and thus directly models the genomic CNV state. For this initial Bayesian analysis of genomic copy number data, we used flat priors for both the CNV start site and width. However, instead of using such noninformative prior, we can assign a prior for the start site inversely proportional to the conservation level of the probe sequence. (This incorporates our belief that the more conserved a sequence is the less likely it is to be duplicated or deleted.) For the width, we can assign the width distribution of known CNVs in the database as a prior. The incorporation of such knowledge through the priors does not need to be done only once: it can be sequential (order-insensitive) as more relevant information becomes available. Using such informative priors, our method can be seen as a framework that enables integration of genomic copy number data and the CNV-related biological knowledge.

## Authors' contributions

ZDZ conceived of the study, implemented the method, performed the analysis, and drafted the manuscript. MBG participated in the design of the study and helped to draft the manuscript. All authors read and approved the final manuscript.
